# The relationship between mechanical power normalized to dynamic lung compliance and weaning outcomes in mechanically ventilated patients

**DOI:** 10.1371/journal.pone.0306116

**Published:** 2024-08-22

**Authors:** Yao Yan, Zhiqiang Du, Haoran Chen, Suxia Liu, Xiaobing Chen, Xiaomin Li, Yongpeng Xie

**Affiliations:** 1 Department of Critical Care Medicine, The Second People’s Hospital of Lianyungang City, Affiliated to Kangda College of Nanjing Medical University, Lianyungang, Jiangsu, China; 2 Department of Emergency Medicine, Lianyungang Clinical College of Nanjing Medical University, The First People’s Hospital of Lianyungang City, Lianyungang, Jiangsu, China; 3 Kangda College of Nanjing Medical University, Lianyungang, Jiangsu, China; 4 Department of Critical Care Medicine, Lianyungang Clinical College of Nanjing Medical University, The First People’s Hospital of Lianyungang City, Lianyungang, Jiangsu, China; Sapienza University of Rome: Universita degli Studi di Roma La Sapienza, ITALY

## Abstract

**Background:**

Prolonged mechanical ventilation is associated with an increased risk of mortality in these patients. However, there exists a significant clinical need for novel indicators that can complement traditional weaning evaluation methods and effectively guide ventilator weaning.

**Objectives:**

To investigate the specific relationship between mechanical power normalized to dynamic lung compliance (Cdyn-MP) and weaning outcomes in patients on mechanical ventilation for more than 24 hours, as well as those who underwent a T-tube weaning strategy.

**Methods:**

A retrospective cohort study was conducted using the Medical Information Mart for Intensive Care-IV v1.0 database (MIMIC-IV v1.0). Patients who received invasive mechanical ventilation for more than 24 hours and underwent a T-tube ventilation strategy for weaning were enrolled. Patients were divided into two groups based on their weaning outcome: weaning success and failure. Ventilation parameter data were collected every 4 hours during the first 24 hours before the first spontaneous breathing trial (SBT).

**Results:**

Of all the 3,695 patients, 1,421 (38.5%) experienced weaning failure. Univariate logistic regression analysis revealed that the risk of weaning failure increased as the Cdyn-MP level rose (OR 1.34, 95% CI 1.31–1.38, P<0.001). After adjusting for age, body mass index, disease severity, and pre-weaning disease status, patients with high Cdyn-MP quartiles in the 4 hours prior to the SBT had a significantly greater risk of weaning failure than those with low Cdyn-MP quartiles (odds ratio 10.37, 95% CI 7.56–14.24). These findings were robust and consistent in both subgroup and sensitivity analyses.

**Conclusion:**

The increased Cdyn-MP before SBT was independently associated with a higher risk of weaning failure in mechanically ventilated patients. Cdyn-MP has the potential to be a useful indicator for guiding the need for ventilator weaning and complementing traditional weaning evaluation methods.

## Introduction

Mechanical ventilation is a crucial life support modality for patients with respiratory failure admitted to the intensive care unit (ICU). Prolonged mechanical ventilation can increase the risk of mortality in these patients [1]. Therefore, timely weaning from the ventilator is essential once the patient’s overall condition has improved. Despite being the most commonly used method for determining ventilator weaning, up to 30% of patients who pass a spontaneous breathing trial (SBT) require reintubation due to weaning failure [2]. The failure of SBTs may be attributed to their inability to accurately reflect airway and lung function over short periods, as well as the lack of objectivity in assessing respiratory muscle endurance during spontaneous breathing load. Therefore, there is a significant clinical need for new indicators that can complement traditional weaning evaluation methods and guide ventilator weaning.

Weaning failure involves complex pathophysiological mechanisms, but the imbalance between respiratory load and respiratory muscle function is considered the primary cause [3, 4]. Mechanical power (MP) refers to the amount of energy transferred from the ventilator to the entire respiratory system per unit time, and it integrates all factors affecting the energy load of the respiratory system, such as pressure, tidal volume (VT), flow rate, and respiratory frequency [5]. MP reflects the respiratory load needed to maintain optimal alveolar ventilation during mechanical ventilation and can help estimate the potential workload of respiratory muscles during the transition to spontaneous breathing [6]. Normalizing MP to dynamic lung compliance, which serves as a surrogate for lung size, may better reflect the anatomical or physiological characteristics unique to each patient and better meet the need for individualized weaning.

Research has indicated that MP normalized to dynamic lung compliance (Cdyn-MP) is independently linked with prolonged mechanical ventilation after tracheotomy and can help identify patients at high risk of weaning failure [7]. However, the specific relationship between Cdyn-MP and weaning outcomes in all patients who receive mechanical ventilation, including those receiving endotracheal intubation, remains unclear. This study aims to investigate the association between Cdyn-MP and weaning outcomes using data from the MIMIC-IV 1.0 database.

## Materials and methods

### Data source

This retrospective study utilized data from the Medical Information Mart for Intensive Care-IV v1.0 database (MIMIC-IV v1.0) [8]. The MIMIC-IV v1.0 database provides comprehensive and high-quality data on well-defined patients admitted to the ICU of Beth Israel Deaconess Medical Center from 2008 to 2019. One of the authors (YY), with certification number 41699414, was responsible for data extraction after obtaining access to the database. All methods employed in this study were in compliance with relevant guidelines and regulations. The data utilized from the MIMIC-IV database were de-identified, and the Institutional Review Boards of the Massachusetts Institute of Technology and Beth Israel Deaconess Medical Center approved the use of the database for research purposes.

### Study cohort

We screened all admissions in the MIMIC-IV database and applied the following inclusion criteria: (1) patients aged 18 years or older at the time of ICU admission; (2) receiving invasive mechanical ventilation (IMV) for more than 24 hours; and (3) first-time ICU admission. Exclusion criteria included: (1) missing SBT records, or not having first-time SBT or extubation records; (2) patients with incomplete ventilation data necessary to calculate MP; and (3) patients not receiving the T-piece ventilation strategy during SBT. The selected patients were then classified into either a weaning success group or a weaning failure group based on their respective weaning outcomes.

### Data collection

We extracted data from the database using Structured Query Language via pgAdmin 4.3. The following variables were extracted from our dataset: (1) basic demographic information such as age, gender, and body mass index (BMI); (2) illness severity, which was assessed at ICU admission using both the Sequential Organ Failure Assessment (SOFA) score and the Simplified Acute Physiology Score II (SAPS II); (3) comorbidities and combined symptoms, identified through International Classification of Diseases, Ninth Revision (ICD-9) codes documented in the MIMIC-IV database; (4) respiratory mechanics parameters recorded within the first 24 hours before SBT, including VT, respiratory rate (RR), positive end-expiratory pressure (PEEP), plateau pressure (Pplat), peak inspiratory pressure (Ppeak), and inspired oxygen fraction (FiO_2_); (5) laboratory indicators before SBT; (6) duration of physiological variables during SBT; and (7) clinical outcomes such as weaning success, duration of IMV, ICU length of stay, 28-day reintubation, and 28-day mortality. Respiratory mechanics parameters were recorded, on average, every 4-hour interval during the 24 hours leading up to the first SBT. For analysis purposes, data from the last 4-hour interval preceding the SBT was used, as it was closest to the weaning process.

### Calculation of Cdyn-MP

We excluded patients with missing data required to calculate MP. Only patients with available Pplat measurements obtained in the volume-controlled model before the SBT were included. We utilized the simplified MP equation proposed by Gattinoni in the volume-controlled model to extract data, using the following formula [5]: MP (J/min) = 0.098 × VT × RR × (Ppeak—0.5 × ΔP), where VT refers to tidal volume, RR to respiratory rate, Ppeak to peak inspiratory pressure, and ΔP to driving pressure. The driving pressure (ΔP) during ventilation was calculated using the following formula: ΔP (cmH_2_O) = Pplat—PEEP, where Pplat represents plateau pressure and PEEP is positive end-expiratory pressure.

The mechanical power normalized to dynamic lung compliance (Cdyn-MP) was calculated using the following formula: Cdyn-MP (J/min×cmH_2_O/ml×10^−3^) = MP / Cdyn. Cdyn measures the compliance of the entire respiratory system and is defined as the change in lung volume resulting from a unit pressure variation [9]. It is calculated as follows: Cdyn (ml/cmH_2_O) = VT / (Ppeak—PEEP), where (Ppeak—PEEP) corresponds to the dynamic driving pressure (ΔPaw).

### Definition of weaning failure

In terms of the definition of SBT, this test involves conducting a trial where a T-tube or low-level pressure support is used in a ventilation model to assess the patient’s capacity to breathe independently without mechanical ventilation assistance. In this study, only patients who were undergoing weaning from T-tube ventilation were included to reduce the impact of diverse SBT modalities on the weaning outcomes [10]. Based on the definition, weaning failure is indicated by premature termination of SBT, necessitating re-intubation or noninvasive ventilation within 48 hours after ending mechanical ventilation, or resulting in mortality within 48 hours after extubation [11]. The criteria for terminating SBT early, as documented in the MIMIC-IV database, consisted of respiratory rate (RR) exceeding 35 breaths/minute for more than 5 minutes, a heart rate (HR) above 140 beats/minute, blood pressure outside the range of 90–180 mmHg, new onset of arrhythmia, SpO_2_ levels dropping below 90% for over 2 minutes, and usage of accessory respiratory muscles [12]. SBT was ceased after clinicians at the bedside observed that the patient’s vital signs exceeded the aforementioned criteria.

### Statistical analysis

Patient characteristics were analyzed based on quartiles of Cdyn-MP. For continuous variables with normal or skewed distributions, data were reported as mean ± standard deviation or median and interquartile range (IQR), respectively. Categorical variables were presented as number (percentage). Student’s t-test or Kruskal-Wallis test was used for group comparison of continuous variables, and chi-square test or Fisher’s exact test was used for categorical variables, as appropriate. Variables with missing data exceeding 25% (S1 Table in the S1 File) were excluded, and multiple imputation was used for data imputation [13].

Univariate and multivariate logistic regression analyses were performed to investigate the association between Cdyn-MP and weaning outcome. Covariates with significant variables (P<0.1) in the univariate analysis and without colinearity were included in the multivariate analysis. Three models were used in the regression analysis: Model 1 adjusted for age, BMI, and SOFA score; Model 2 adjusted for Model 1 plus RR, PEEP, Pplat, and FiO_2_; and Model 3 adjusted for Model 2 plus white blood cell (WBC) count, serum creatinine (SCr), urine output per hour (Uorate), HR, mean blood pressure (MBP), and blood oxygen saturation (SpO_2_) determined using pulse oximetry. To investigate the relationship between Cdyn-MP and weaning failure, we used restricted cubic spline models [14] and accounted for the covariates mentioned in Model 3. The median value of Cdyn-MP was used as the reference point, with knots placed at the 5th, 35th, 65th, and 95th percentiles of Cdyn-MP.

We utilized a stratified linear regression model and likelihood ratio test to detect any modifications and interactions in subgroups based on various factors: age (<65 or ≥65 years), gender (female or male), BMI (<28 or ≥28 kg/cm^2^), SOFA score (<7 or ≥7), smoking (yes or no), and chronic obstructive pulmonary disease (COPD; yes or no). We also conducted additional subgroup analyses by using separate models and treating Cdyn-MP as a categorical variable. In sensitivity analysis, we performed a complete case analysis assuming data was missing completely at random. To investigate the relationship between Cdyn-MP and weaning outcome, an additional sensitivity analysis was conducted on mechanically ventilated patients with diverse comorbidities. We used a two-tailed test and considered P<0.05 as statistically significant. All statistical analyses were performed using R statistical software (version 4.1.2; http://www.R-project.org; The R Foundation) and Free Statistics (version 1.7).

## Results

**Fig 1:**
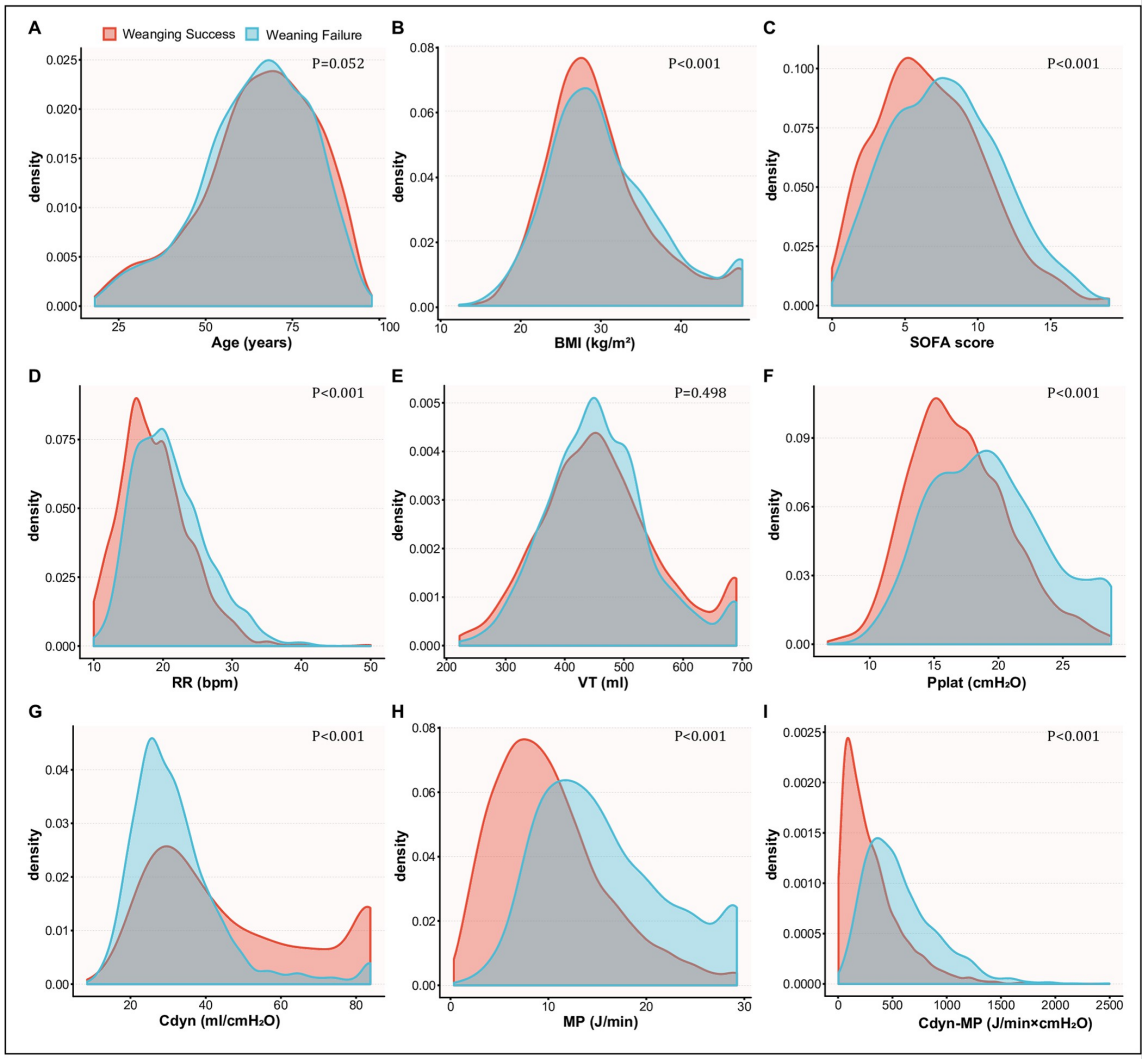
Distribution of baseline characteristics and ventilatory variables between the weaning success group and the weaning failure group. (A) Age. (B) Body mass index (BMI). (C) SOFA score. (D) Respiratory rate (RR). (E) Tidal volume (VT). (F) Plateau pressure (Pplat). (G) Dynamic lung compliance (Cdyn). (H) Mechanical power (MP). (I) MP normalized to Cdyn (Cdyn-MP). The area shaded with red shows the density map of the frequency distribution of weaning success group, and blue represents the weaning failure group. In the depicted figure, the weaning failure group exhibited higher values for the BMI, SOFA score, RR, Pplat, Cdyn, MP, and Cdyn-MP, as compared to the weaning success group.

We conducted a retrospective analysis on 76,540 hospitalized patients whose data were recorded in the MIMIC-IV v1.4 database. Out of those patients, 3,695 received invasive ventilation for at least 24 hours and were weaned using the T-tube ventilation strategy. Among the 3,695 patients, 2,274 achieved successful weaning, while 1,421 experienced weaning failure. Within 48 hours of the first successful SBT, 11.1% (283/2,557) of patients required reintubation or non-invasive mechanical ventilation, or experienced mortality. S1 Fig in the S1 File illustrates the screening process. The distribution of baseline characteristics and ventilatory variables between the weaning success group and the weaning failure group are shown in Fig 1.

Out of the 3,695 patients analyzed, the median age was 66.8 years (IQR: 55.4–77.5 years), and 2,102 (56.9%) were male. The Cdyn-MP before SBT at 4 hours was 331.9 J/min×cmH_2_O/ml×10^−3^ (IQR: 164.7–562.2 J/min×cmH_2_O/ml×10^−3^), and the median duration of IMV was 3.7 days (IQR 1.8–8.1 days). Weaning failure occurred in 38.5% of patients. Additional details are provided in Table 1.

**Table 1:** Clinical characteristics of the study population according to mechanical power normalized to dynamic lung compliance (Cdyn-MP).

Variables	Cdyn-MP					P value
	Total (n = 3695)	Q1 (n = 924)	Q2 (n = 923)	Q3 (n = 924)	Q4 (n = 924)	
		(≤164.7)	(164.7–331.6)	(331.6–561.9)	(≥561.9)	
Age (years)	66.8 (55.4–77.5)	66.5 (55.4–76.6)	67.9 (55.8–79.4)	67.8 (56.6–78.5)	65.2 (54.1–75.0)	< 0.001
Gender (male)	2102 (56.9)	534 (57.8)	527 (57.1)	494 (53.5)	547 (59.2)	0.080
BMI (kg/m^2^)	27.9 (24.5–32.6)	27.4 (24.2–31.4)	26.9 (24.0–30.8)	28.2 (24.6–33.2)	29.5 (25.6–35.3)	< 0.001
SOFA score	7.0 (4.0–10.0)	6.0 (4.0–9.0)	7.0 (4.0–9.0)	7.0 (5.0–10.0)	8.0 (6.0–11.0)	< 0.001
SAPS II	43.0 (34.0–53.0)	40.0 (31.0–50.0)	42.0 (33.5–52.0)	44.0 (35.0–54.0)	45.0 (37.0–57.0)	< 0.001
Smoking, n (%)	315 (8.5)	86 (9.3)	65 (7)	77 (8.3)	87 (9.4)	0.230
Comorbidities, n (%)						
Hypertension	1479 (40.0)	370 (40)	366 (39.7)	400 (43.3)	343 (37.1)	0.060
Diabetes	1138 (30.8)	276 (29.9)	252 (27.3)	304 (32.9)	306 (33.1)	0.019
COPD	256 (6.9)	61 (6.6)	65 (7.0)	57 (6.2)	73 (7.9)	0.503
Congestive heart failure	1107 (30.0)	228 (24.7)	281 (30.4)	297 (32.1)	301 (32.6)	< 0.001
Chronic kidney disease	838 (22.7)	185 (20)	200 (21.7)	219 (23.7)	234 (25.3)	0.037
Stroke	733 (19.8)	192 (20.8)	207 (22.4)	189 (20.5)	145 (15.7)	0.002
Respiratory mechanics parameters before SBT 4h						
VT (ml)	451 (395–512)	478 (398–567)	442 (388–500)	448 (396–500)	450 (397–500)	< 0.001
RR (bpm)	19 (16–22)	16.5 (14–20)	18 (16–21)	19 (16–22)	23 (20–26)	< 0.001
PEEP (cmH_2_O)	5.0 (5.0–8.0)	5.0 (3.3–5.0)	5.0 (5.0–5.5)	5.1 (5.0–8.0)	8.0 (5.0–11.0)	< 0.001
Pplat (cmH_2_O)	17.5 (15.0–20.5)	15.5 (13.0–19.0)	15.0 (14.0–18.0)	18.0 (16.0–20.0)	21.0 (19.0–24.0)	< 0.001
Peak (cmH_2_O)	19.0 (15.0–24.0)	12.0 (11.0–14.0)	17.0 (16.0–19.0)	21.3 (20.0–23.0)	27.0 (24.0–30.0)	< 0.001
ΔPaw (cmH_2_O)	13.0 (9.4–16.5)	7.0 (6.0–9.0)	11.5 (10.0–13.0)	15.0 (13.0–17.0)	18.4 (16.0–21.0)	< 0.001
Cdyn (ml/cmH_2_O)	33.9 (26.2–49.2)	67.9 (51.3–83.8)	38.5 (32.2–45.9)	29.9 (25.5–34.8)	24.2 (20.5–28.3)	< 0.001
MP (J/min)	11.1 (7.4–16.1)	5.3 (3.6–7.0)	9.4 (7.8–11.4)	12.8 (10.8–15.3)	19.9 (16.3–24.6)	< 0.001
Cdyn-MP (J/min×cmH_2_O/ml×10^−3^)	331.9 (164.7–562.2)	85.7 (54.6–123.1)	245.5 (203.8–291.1)	426.7 (379.4–496.0)	786.9 (658.3–985.0)	< 0.001
FiO_2_ (%)	40.0 (40.0–50.0)	40.0 (40.0–42.5)	40.0 (40.0–50.0)	40.0 (40.0–50.0)	50.0 (40.0–50.0)	< 0.001
Laboratory data before SBT						
WBC (k/ul)	11.8 (8.8–15.6)	11.3 (8.6–14.5)	11.3 (8.6–15.1)	11.9 (8.8–15.7)	12.8 (9.4–17.1)	< 0.001
PLT (k/ul)	164 (112–223)	158.5 (109–216)	166 (111–221)	165 (116–224)	170 (112–233)	0.095
Hb (g/dl)	9.9 (8.7–11.3)	10.0 (8.8–11.3)	9.9 (8.7–11.4)	9.8 (8.7–11.3)	9.8 (8.6–11.3)	0.332
SCr (mg/dl)	1.1 (0.8–1.8)	1.0 (0.7–1.6)	1.1 (0.7–1.7)	1.1 (0.8–1.8)	1.3 (0.9–2.2)	< 0.001
Uorate before SBT (ml/kg/h)	0.6 (0.4–1.1)	0.7 (0.5–1.2)	0.7 (0.4–1.1)	0.6 (0.3–1.1)	0.5 (0.3–1.0)	< 0.001
Physiological variables during SBT						
Temperature (°C)	37.1 (36.8–37.4)	37.1 (36.8–37.4)	37.1 (36.7–37.4)	37.1 (36.8–37.4)	37.1 (36.8–37.5)	0.082
HR (bpm)	83 (72–97)	82 (72–95)	82 (71–96)	83 (72–96)	87 (75–100)	< 0.001
BF (bpm)	19 (16–22)	17 (14–20)	18 (15–21)	19 (16–22)	22 (19–26.)	< 0.001
MBP (mmHg)	75.0 (68.0–84.0)	76.3 (68.0–86.6)	75.5 (68.0–85.0)	75.0 (67.0–83.0)	74.0 (67.0–82.0)	< 0.001
SPO_2_ (%)	98 (97–100)	99 (97–100)	99 (97–100)	99 (97–100)	97 (96–99)	< 0.001
Clinical outcomes						
Weaning failure, n (%)	1421 (38.5)	70 (7.6)	307 (33.3)	456 (49.4)	588 (63.6)	< 0.001
Duration of IMV (days)	3.7 (1.8–8.1)	2.5 (1.5–4.7)	3.2 (1.6–6.7)	4.0 (2.0–8.5)	6.3 (3.0–10.7)	< 0.001
ICU LOS (days)	6.8 (4.0–11.8)	5.1 (3.4–8.5)	6.2 (3.7–10.5)	7.2 (4.1–12.3)	9.2 (5.7–14.2)	< 0.001
28-day Reintubation, n (%)	531 (14.4)	86 (9.3)	123 (13.3)	148 (16)	174 (18.8)	< 0.001
28-day Mortality, n (%)	693 (18.8)	122 (13.2)	160 (17.3)	196 (21.2)	215 (23.3)	< 0.001

BMI—body mass index, SOFA—sequential organ failure assessment, SAPS II—simplified acute physiology score II, COPD—chronic obstructive pulmonary disease, SBT—spontaneous breathing trial, VT—tidal volume, RR—respiratory rate, PEEP—positive end expiratory pressure, Pplat—plateau pressure, Ppeak—peak inspiratory pressure, ΔPaw—dynamic driving pressure, Cdyn—dynamic lung compliance, MP—mechanical power, Cdyn-MP—MP normalized to Cdyn, FiO_2_—inspired oxygen concentration, WBC—white blood cell count, PLT—platelets, Hb—hemoglobin, SCr—serum creatinine, Uorate—urine output per hour, HR—heart rate, BF—breathing frequency during SBT, MBP—mean blood pressure, SPO_2_—pulse oximetry, IMV—invasive mechanical ventilation, LOS—length of stay.

### Relationship between Cdyn-MP and weaning failure

**Fig 2:**
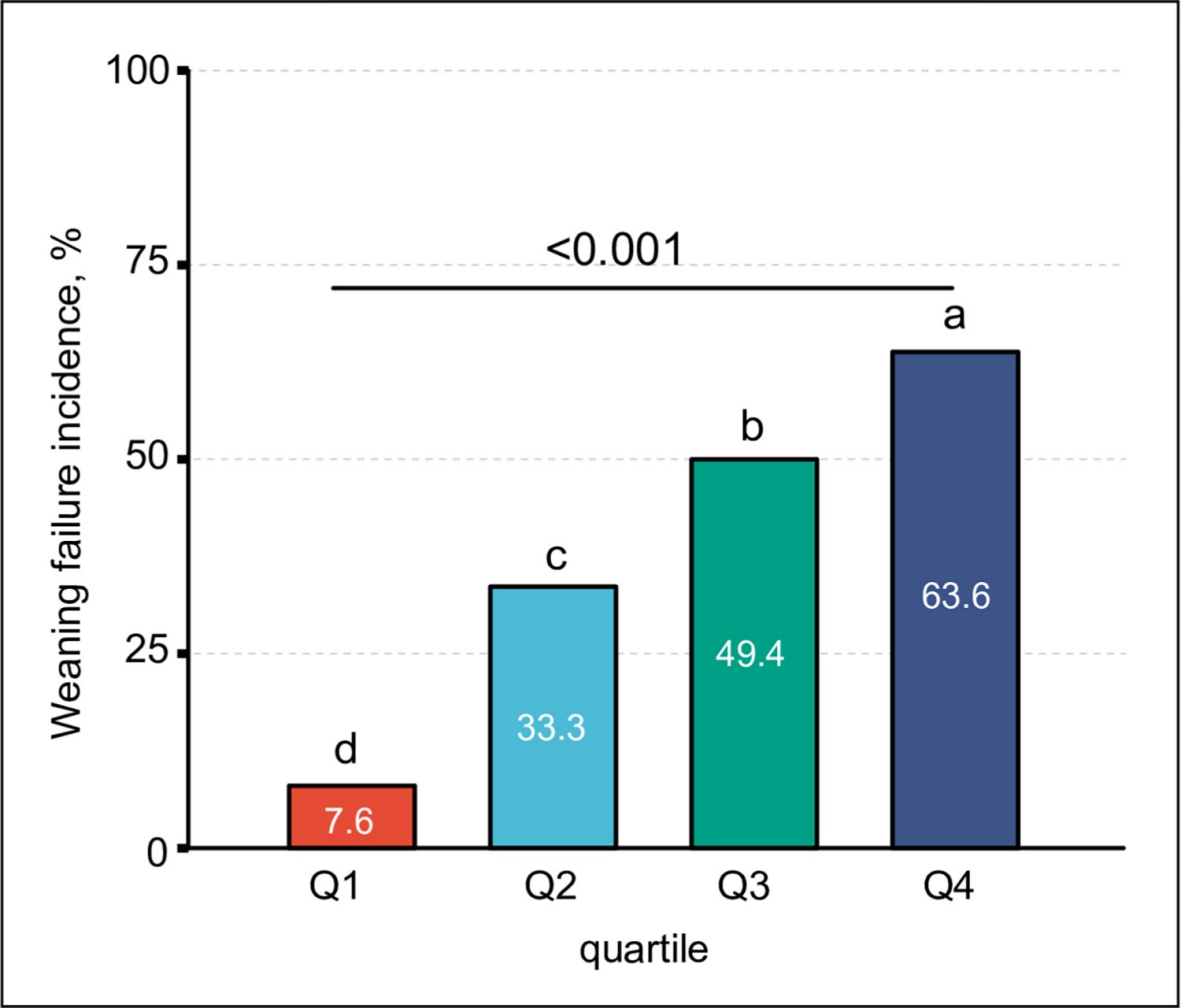
Weaning failure incidence as expressed in MP normalized to dynamic lung compliance (Cdyn-MP) quartile. Q1, Q2, Q3, and Q4 are quartiles of the Cdyn-MP. Corresponding quartile cut-off values are shown in Table 1. Different letters represent significant differences between different groups (P<0.05).

The total incidence of weaning failure in patients on IMV was 38.5% (1421/3695). The incidence increased from 7.6% in the first Cdyn-MP quartile to 63.6% in the fourth Cdyn-MP quartile (P<0.001, Fig 2).

The results of the univariate logistic regression analysis for weaning outcome are presented in S2 Table in the S1 File. When Cdyn-MP was expressed as a continuous variable in the logistic regression analysis, it was found to have a significant association with weaning failure (odds ratio [OR] 1.34; 95% confidence interval [CI] 1.31–1.38; P<0.001; refer to Table 2, Model 1). This association remained statistically significant even after adjusting for potential confounding factors, including age, BMI, SOFA score, RR, PEEP, Pplat, FiO_2_, WBC count, SCr, urate, HR, MBP, and SpO_2_, with an odds ratio of 1.24 (95% CI 1.20–1.29; P < 0.001; see Table 2, Model 3).

**Table 2:** Multivariable-adjust odds ratio and 95% confidence interval of the Cdyn-MP quartiles associated with weaning failure.

Variables	Unadjusted		Model 1		Model 2		Model 3	
	OR (95% CI)	P value	OR (95% CI)	P value	OR (95% CI)	P value	OR (95% CI)	P value
Cdyn-MP (per100J/min×cmH_2_O/ml×10^−3^)	1.34 (1.31–1.38)	< 0.001	1.33 (1.30–1.37)	< 0.001	1.24 (1.20–1.28)	< 0.001	1.24 (1.20–1.29)	< 0.001
Q1 (≤164.7)	1 (Ref)		1 (Ref)		1 (Ref)		1 (Ref)	
Q2 (164.7–331.6)	6.08 (4.60–8.04)	< 0.001	6.04 (4.56–7.99)	< 0.001	5.03 (3.78–6.69)	< 0.001	5.05 (3.80–6.72)	< 0.001
Q3 (331.6–561.9)	11.89 (9.02–15.66)	< 0.001	11.67 (8.85–15.39)	< 0.001	8.22 (6.18–10.94)	< 0.001	8.27 (6.21–11.01)	< 0.001
Q4 (≥561.9)	21.35 (16.17–28.2)	< 0.001	20.05 (15.13–26.57)	< 0.001	10.35 (7.54–14.19)	< 0.001	10.37 (7.56–14.24)	< 0.001
P for trend		< 0.001		< 0.001		< 0.001		< 0.001

OR—odds ratio, CI—confidence interval, Cdyn-MP—mechanical power normalized to dynamic lung compliance. Q1, Q2, Q3, and Q4 are quartiles of the Cdyn-MP.

Model 1 was adjusted for age, BMI and SOFA score.

Model 2 was adjusted for Model 1+RR, PEEP, Pplat and FiO_2_.

Model 3 was adjusted for Model 2+WBC, SCr, Uorate, HR, MBP and SpO_2_.

In the multivariate logistic regression analysis, when Cdyn-MP was expressed in quartiles, there was a 10.37 times higher risk of weaning failure in the highest quartile (OR 10.37; 95% CI 7.56–14.24; P<0.001) compared to the lowest quartile, independently of the potential confounders (see Table 2, Model 3). Compared with the lowest Cdyn-MP group Q1 (≤164.7 J/min×cmH_2_O/ml×10^−3^), the adjusted OR values for Cdyn-MP and weaning failure in Q2 (164.7–331.6 J/min×cmH_2_O/ml×10^−3^), Q3 (331.6–561.9 J/min×cmH_2_O/ml×10^−3^) and Q4 (≥561.9 J/min×cmH_2_O/ml×10^−3^) were 5.05 (95% CI 3.80–6.72, P<0.001), 8.27 (95% CI 6.21–11.01, P<0.001), 10.37 (95% CI 7.56–14.24, P<0.001), respecitively (see Table 2, Model 3).

**Fig 3:**
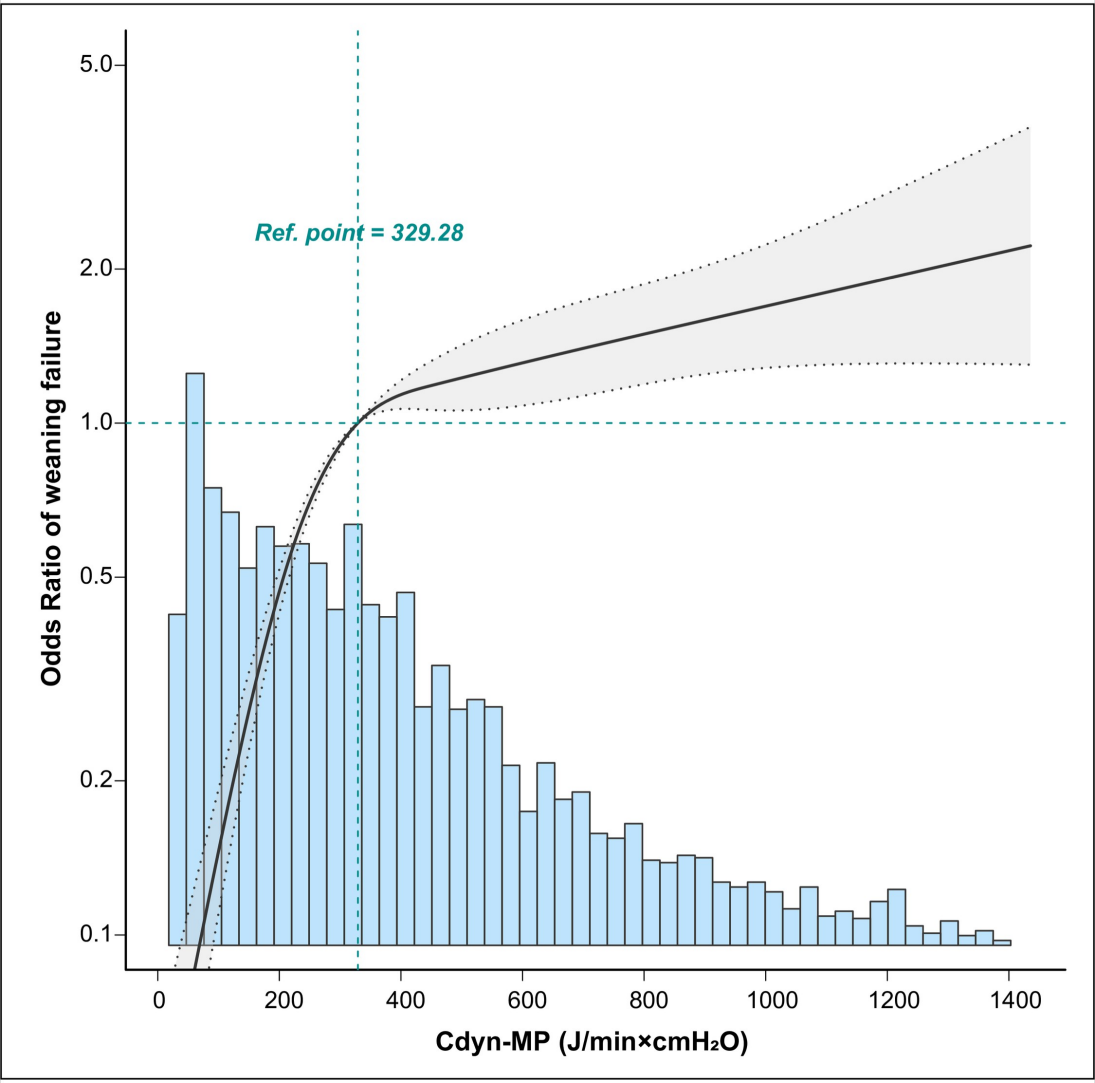
Association between mechanical power normalized to dynamic lung compliance (Cdyn-MP) and weaning failure. Solid lines represent the best-fit curve, and dashed lines represent a 95% confidence interval. Odd ratios (ORs) were adjusted for age, body mass index, SOFA score, respiratory rate, positive end-expiratory pressure, plateau pressure, FiO_2_, white blood cell count, serum creatinine, Uorate, heart rate, mean blood pressure and SpO_2_. The blue histograms represent the frequency distribution for weaning failure among mechanically ventilated patients. Only 99% of the data are shown.

Fig 3 displays a multivariate adjusted restricted cubic spline that illustrates the association between Cdyn-MP and weaning failure. The figure indicates a significant positive correlation between Cdyn-MP and the risk of weaning failure in mechanically ventilated patients.

### Subgroup analyses by adjusted potential effect of confounders

**Fig 4:**
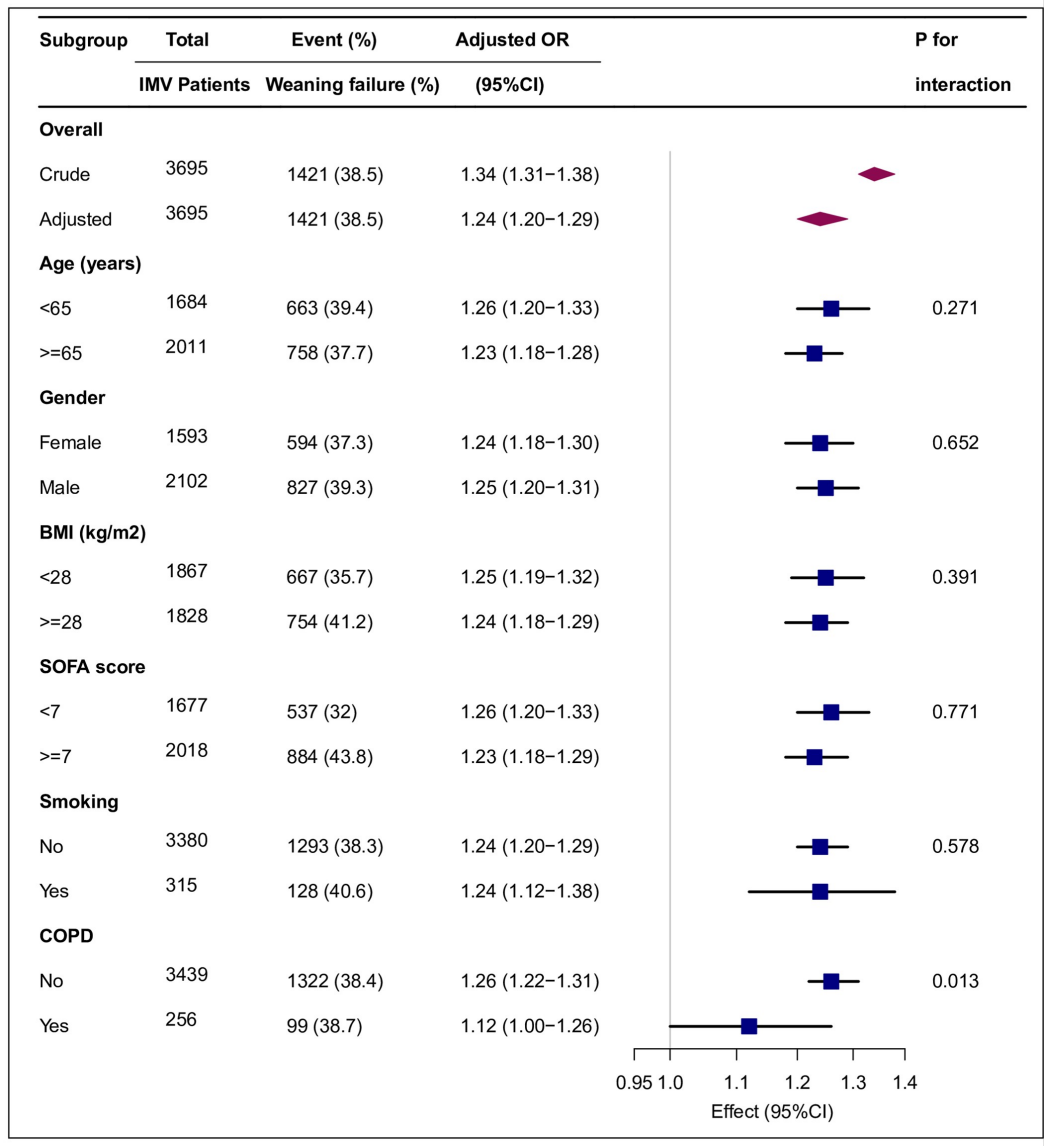
Subgroup analyses of the mechanical power normalized to dynamic lung compliance (Cdyn-MP) and weaning failure. Each stratification was adjusted for age, body mass index (BMI), SOFA score, respiratory rate, positive end-expiratory pressure, plateau pressure, FiO_2_, white blood cell count, serum creatinine, Uorate, heart rate, mean blood pressure and SpO_2_ except the stratification factor itself. Diamonds indicate overall odds ratios (ORs), with outer points of the diamonds. indicating 95% CIs. Squares indicate ORs, with horizontal lines indicating 95% confidence intervals. IMV, invasive mechanical ventilation; COPD, chronic obstructive pulmonary disease.

Subgroup analyses were conducted to assess the impact of Cdyn-MP (per 100-unit increment) on weaning failure in different subgroups, including age, gender, BMI, SOFA score, smoking, and COPD. Fig 4 illustrates the results of these subgroup analyses, where no significant interactions were observed within any subgroup. Furthermore, S2 Fig in the S1 File demonstrates that when analyzing Cdyn-MP as quartiles, the relationship remained consistent and significant.

### Sensitivity analysis and additional analyses

Sensitivity analyses were conducted to assess the robustness of the findings. Firstly, a multivariate logistic regression model was applied to cases with complete data (see S3 Table in the S1 File). Additionally, an extra sensitivity analysis was performed on mechanically ventilated patients with different comorbidities to examine the relationship between Cdyn-MP and weaning failure (see S3 Fig in the S1 File). Importantly, consistent associations were observed in both these sensitivity analyses. The multivariate adjusted restricted cubic spline plots clearly illustrated a gradual escalation in the risk of weaning failure as Cdyn-MP increased among patients with various comorbidities such as congestive heart failure, chronic kidney disease, diabetes, and stroke (S3 Fig in the S1 File). Additional analyses are presented in S4 Table in the S1 File.

## Discussion

Our study demonstrated that increased Cdyn-MP before SBT was associated with a higher risk of weaning failure in mechanically ventilated patients, independent of age, BMI, and disease characteristics. These findings suggest that Cdyn-MP measurements could provide critical insights to guide the ventilation weaning process and help clinicians promote successful extubation, ultimately improving patient outcomes. As a new indicator that complements traditional weaning evaluation methods, Cdyn-MP may prove valuable in facilitating timely and effective weaning from mechanical ventilation.

Optimizing the timing of weaning from mechanical ventilation is crucial in reducing the risk of weaning failure and improving clinical outcomes for critically ill patients in the ICU. The reasons for failed weaning from mechanical ventilation involve multiple aspects, including respiratory, neurological, cardiovascular, diaphragmatic, and endocrine-metabolic factors [3]. However, the primary reasons for weaning failure are increased respiratory load and increased work of respiratory muscles caused by increased airway resistance and decreased respiratory system compliance, accounting for about 60% of all failed weaning cases [15].

In 2016, German scholar Gattinoni introduced the concept of mechanical power (MP) to assess ventilator-induced lung injury in ARDS patients [5]. MP measures the impact of various mechanical parameters–such as RR, VT, flow rate, and PEEP–on the total power delivered by the ventilator. These variables are aggregates into a single physical measure that reflects the overall energy load imposed on the respiratory muscles during mechanical ventilation. As a comprehensive respiratory mechanics index, MP has emerged as a valuable tool for assessing respiratory function in clinical practice and quantifying the energy required to overcome lung resistance and maintain optimal oxygenation during mechanical ventilation [16, 17]. Ghiani et al. reported that MP is a crucial determinant of sufficient gas exchange in the body and a key factor in evaluating a patient’s potential for successful weaning from mechanical ventilation [7, 9].

However, because of the presence of various factors that lead to the uneven distribution of energy in the lungs, such as the size and inhomogeneity of the lungs, the extent of stress risers, and the vessels’ filling state, the same MP before weaning may have different effects on different individuals [16]. MP is associated with the size of the functional lung volume, and its diagnostic accuracy can be improved by normalizing for lung size substitutes, such as well-inflated lung tissue determined by computed tomography analysis, lung compliance during ventilation, or predicted body weight [5, 18–20]. Therefore, Cdyn-MP is a better representation of the actual energy transmitted from the respiratory muscles to the lungs, and it can better reflect the pathological and physiological status of the lungs before weaning in different critically ill patients in the ICU setting, which may have a better guiding value for weaning outcome.

In this study, we found a positive correlation between Cdyn-MP and the risk of weaning failure in mechanically ventilated patients. After adjusting for confounding factors, Cdyn-MP remained independently associated with weaning failure (OR 1.24; 95% CI 1.20–1.29). Patients in the high-Cdyn-MP-quartile group had a 10.37 times higher risk of weaning failure compared to those in the low-quartile group (Table 2). This association was consistent across various subgroups, as no significant interactions were observed when stratifying by age, gender, BMI, SOFA score, smoking, and COPD. The sensitivity analysis results confirmed the association between Cdyn-MP and weaning failure in both complete data and IMV patients with different comorbidities. Higher Cdyn-MP values may correlate with weaning failure due to potential lung damage or pre-existing lung conditions requiring increased ventilatory support, such as elevated peak pressure, PEEP, and respiratory rate. In addition, Cdyn-MP demonstrated superior predictive performance for weaning outcomes compared to several other related parameters, including PEEP, dynamic driving pressure, and dynamic lung compliance (S4 Table in the S1 File). This underscores its potential as a valuable tool for preweaning evaluation.

To our knowledge, only two studies have examined the correlation between MP normalized to lung compliance and weaning outcome at a specialized national weaning center in Germany. Ghiani et al. conducted a retrospective observational study on 263 prolonged tracheotomized patients undergoing mechanical ventilation at the center. They found that MP standardized to dynamic lung-thorax compliance (LTC-MP) was independently associated with weaning outcome and could help identify patients at high risk for weaning failure [4]. Furthermore, the authors demonstrated the superior predictive power of LTC-MP compared to MP in forecasting weaning outcome through another prospective study [7]. They found that LTC-MP could accurately predict the risk of weaning failure. However, the previous study’s findings were restricted to tracheotomized patients who had undergone mechanical ventilation for over three weeks. Our current study, based on data from the MIMIC-IV database, identified a correlation between Cdyn-MP and weaning outcome in all patients receiving mechanical ventilation, including those with endotracheal intubation. These findings are in line with Ghiani et al.’s research, providing additional evidence that supports and enhances the clinical relevance of their findings.

There are some limitations to this study, which should be considered. Firstly, as with any regression analysis, residual confounders may still exist. Through subgroup and sensitivity analyses, we attempted to adjust for possible confounders and minimize the influence of factors that may lead to outcome bias. Secondly, it should be noted that this study is a secondary analysis of the dataset in MIMIC-IV, which was originally collected for clinical purposes. As a result, there cannot be an absolute guarantee that the parameters were obtained under strictly controlled conditions, such as absence of spontaneous breathing and adequate sedation. Nonetheless, we followed the methodology proposed by Serpa Neto A et al. [21] for extracting respiratory parameters, and rigorous quality control measures were implemented to enhance the accuracy and reliability of the data. Thirdly, we extracted data and calculated mechanical power according to the simplified MP equation proposed by Gattinoni in the volume-controlled ventilation mode in our study. Patients who did not have a Pplat measurement under volume-controlled ventilation were excluded from the analysis. Further investigation may be required to explore this relationship in pressure-controlled ventilation mode.

## Conclusions

Cdyn-MP is an independent predictor of weaning outcome in mechanically ventilated patients, independent of age, BMI, and disease characteristics. Cdyn-MP can better reflect the pathological and physiological status of the lungs of different patients on mechanical ventilation before weaning. By serving as a quantitative reference index for pre-weaning respiratory load, Cdyn-MP has the potential to be a valuable tool for guiding the decision to wean from mechanical ventilation.

## Supporting information

S1 Checklist Human subjects research checklist. (DOCX)

S1 File Supplementary methods, tables, and figures. (DOCX)
