# Supplementary material for: The relationship between mechanical power normalized to dynamic lung compliance and weaning outcomes in mechanically ventilated patients
Source: PLoS One. 2024 Aug 22;19(8):e0306116. doi: 10.1371/journal.pone.0306116 (PMC11341020; doi:10.1371/journal.pone.0306116)

***Supplementary materials***

**1.** **Supplementary methods** (Page 2-4)

**2. S1 Table.** **Percentage of missing data in variables of interest at baseline.** (Page 5)

**3.** **S2 Table.** **Univariate logistic regression analysis of risk factors for weaning failure.** (Page 6)

**4.** **S3 Table.** **Association between Cdyn-MP and weaning failure in complete cases.** (Page 7)

**5.** **S4 Table.** **Predictability of related parameters for weaning failure occurrence in mechanically ventilated patients.** (Page 8)

**6.** **S1 Fig.** **Flowchart showing the screening process used in the study.** (Page 9)

**7.** **S2 Fig.** **Relationship between Cdyn-MP and weaning failure in patients on mechanical ventilation according to basic features.** (Page 10)

**8.** **S3 Fig.** **Association between mechanical power normalized to dynamic lung compliance (Cdyn-MP) and weaning failure in mechanically ventilated patients with different comorbidities.** (Page 11)

**Supplementary methods**

**Approach to missing data**

Missing data are summarized in **S1** **Table**. Due to a high percentage (greater than 30%) of missing values, variables such as albumin and arterial blood gas analysis during SBT (PH, PaCO_2_, PaO_2_, PaO_2_/FiO_2_) were excluded from the study. Variables with less than 25% missing data were imputed through multiple imputation using the statistical software R (version 4.1.2; [http://www.R-project.org](http://www.r-project.org/); The R Foundation).

**Subgroup analyses**

We further performed a stratified analysis based on quartiles of MP normalized to dynamic lung compliance (Cdyn-MP). After stratifying by age, gender, BMI, SOFA score, smoking, and COPD, we did not find any significant interactions in any subgroups (see **S2 Fig**). Notably, a similar association was observed between Cdyn-MP and weaning failure in patients receiving mechanical ventilation. The **S2 Fig** clearly showed that, except for the COPD population, there was a significant association between increasing Cdyn-MP quartiles and a higher risk of weaning failure.

**Sensitivity analyses**

To perform sensitivity analyses, we repeated the analyses on cases with complete data (n=2,078) without the need for imputing missing data. We found no significant differences between cases with complete data and those with imputed data. Moreover, the association between Cdyn-MP and weaning failure remained unchanged in multivariate models (OR 1.23, 95% CI 1.18-1.28, P<0.001; see **S3 Table**, model 3). After adjusting for all relevant covariates, we confirmed that these associations were still significant when Cdyn-MP was treated as a categorical variable (P<0.001; see **S3 Table**, model 3).

We also performed an extra sensitivity analysis for further verification of the association between Cdyn-MP and weaning failure in mechanically ventilated patients with different comorbidities (see **S3 Fig**). Importantly, consistent associations were observed in both these sensitivity analyses. The multivariate adjusted restricted cubic spline plots clearly illustrated a gradual escalation in the risk of weaning failure as Cdyn-MP increased among patients with various comorbidities such as congestive heart failure, chronic kidney disease, diabetes, and stroke (**S3 Fig**).

**Predictive value of related parameters**

We evaluated the predictive value of PEEP, dynamic driving pressure (Δpaw), dynamic lung compliance (Cdyn), MP, and Cdyn-MP for weaning failure using receiver operating characteristic (ROC) analysis (**S4 Table**). Cdyn-MP had an area under the ROC curve (AUC) of 0.760 (95% CI 0.745-0.776, P<0.001) for the prediction of weaning failure. The optimal cutoff value for Cdyn-MP was determined to be 317.8 J/min×cmH_2_O/ml×10^−3^, with a sensitivity of 76% and a specificity of 63%. In comparison, the AUCs for PEEP, ΔPaw, Cdyn, and MP were 0.712 (95% CI 0.695-0.729, P<0.001), 0.667 (95% CI 0.650-0.684, P<0.001), 0.689 (95% CI 0.672-0.706, P<0.001), and 0.745 (95% CI 0.730-0.761, P<0.001), respectively. These results suggest that Cdyn-MP performs better than the other respiratory mechanics parameters in predicting weaning outcomes, as indicated by the higher AUC value (**S4 Table**).

**S1 Table. Percentage of missing data in variables of interest at baseline.**

| **Variables** | **n (%)** |
| --- | --- |
| Age (years) | 0 (0%) |
| Gender (male) | 0 (0%) |
| BMI (kg/m^2^) | 782 (21.2%) |
| SOFA score | 0 (%) |
| SAPS II | 0 (%) |
| VT (ml) | 0 (%) |
| RR (bpm) | 0 (%) |
| PEEP (cmH_2_O) | 0 (%) |
| Pplat (cmH_2_O) | 0 (%) |
| Peak (cmH_2_O) | 0 (%) |
| ΔPaw (cmH_2_O) | 0 (%) |
| Cdyn (ml/cmH_2_O) | 0 (%) |
| MP (J/min) | 0 (%) |
| Cdyn-MP (J/min×cmH_2_O) | 0 (%) |
| FiO_2_ (%) | 61 (1.65%) |
| WBC (k/ul) | 404 (10.9%) |
| PLT (k/ul) | 333 (9.0%) |
| Hb (g/dl) | 310 (8.4%) |
| Albumin (g/dl) | 1708 (46.2%) |
| SCr (mg/dl) | 346 (9.4%) |
| Uorate before SBT (ml/kg/h) | 112 (3.0%) |
| Temperature (℃) | 344 (9.3%) |
| PH | 2074 (56.1%) |
| PaO_2_ (mmHg) | 2046 (55.4%) |
| PaCO_2_ (mmHg) | 2079 (56.3%) |
| PaO_2_/FiO_2_ (mmHg) | 2090 (56.6%) |
| HR (bpm) | 0 (0%) |
| BF (bpm) | 0 (0%) |
| MBP (mmHg) | 0 (0%) |
| SPO_2_ (%) | 0 (0%) |

BMI - body mass index, SOFA - sequential organ failure assessment, SAPS II - simplified acute physiology score II, VT - tidal volume, RR - respiratory rate, PEEP - positive end expiratory pressure, Pplat - plateau pressure, Ppeak - peak inspiratory pressure, ΔPaw - dynamic driving pressure, Cdyn - dynamic lung compliance, MP - mechanical power, Cdyn-MP - MP normalized to dynamic lung compliance, FiO_2_ - inspired oxygen concentration, WBC - white blood cell count, PLT - platelets, Hb - hemoglobin, SCr - serum creatinine, SBT - spontaneous breathing trial, PaO_2_ - partial pressure of oxygen, PaCO_2_ - arterial partial pressure of carbon dioxide, PaO_2_/FiO_2_ - oxygenation indexarterial - (i.e. - PaO_2_ divided by the FiO_2_), HR - heart rate, BF - breathing frequency during SBT, MBP - mean blood pressure, SPO_2_ - pulse oximetry.

| **S2 Table. Univariate logistic regression analysis of risk factors for weaning failure.** | | | | | | | |  |
| --- | --- | --- | --- | --- | --- | --- | --- | --- |
| **Variables** | **OR (95% CI)** | ***P* value** |  | **Variables** | **OR (95% CI)** | | ***P* value** | |
| Age (years) | 1.00 (0.99-1.00) | 0.108 | Peak (cmH_2_O) | | | 1.16 (1.14-1.17) | <0.001 |  |
| Gender (male) | 1.09 (0.95-1.25) | 0.203 | ΔPaw (cmH_2_O) | | | 1.12 (1.11-1.14) | <0.001 |  |
| BMI (kg/m^2^) | 1.02 (1.01-1.03) | 0.001 | Cdyn (ml/cmH_2_O) | | | 0.96 (0.95-0.96) | <0.001 |  |
| SOFA score | 1.08 (1.06-1.10) | <0.001 | MP (J/min) | | | 1.15 (1.14-1.16) | <0.001 |  |
| SAPS II | 1.01 (1.01-1.02) | <0.001 | Cdyn-MP (per100J/min ×cmH_2_O/ml×10^−3^) | | | 1.34 (1.31-1.38) | <0.001 |  |
| Smoking | 1.10 (0.87-1.40) | 0.406 | FiO_2_ (%) | | | 1.06 (1.06-1.07) | <0.001 |  |
| Comorbidities | | | Laboratory data at the start of SBT | | | | |  |
| Hypertension | 1.04 (0.91-1.19) | 0.571 | WBC (per 100 k/ul) | | | 1.03 (1.02-1.05) | <0.001 |  |
| Diabetes | 1.09 (0.94-1.25) | 0.261 | PLT (k/ul) | | | 1.06 (0.99-1.15) | 0.108 |  |
| COPD | 1.01 (0.78-1.31) | 0.942 | Hb (g/dl) | | | 1.03 (1.00-1.07) | 0.065 |  |
| Congestive heart failure | 1.03 (0.90-1.20) | 0.643 | SCr (mg/dl) | | | 1.18 (1.09-1.27) | <0.001 |  |
| Chronic kidney disease | 1.02 (0.87-1.19) | 0.826 | Uorate before SBT (ml/kg/h) | | | 0.79 (0.71-0.89) | <0.001 |  |
| Stroke | 1.09 (0.92-1.29) | 0.305 | Physiological variables during SBT | | | | |  |
| Respiratory mechanics parameters before SBT 4h | | | Temperature (℃) | | | 1.06 (0.95-1.18) | 0.307 |  |
| VT (per 100 ml) | 0.96 (0.89-1.02) | 0.188 | HR (bpm) | | | 1.01 (1.00-1.01) | <0.001 |  |
| RR (bpm) | 1.09 (1.07-1.10) | <0.001 | BF (bpm) | | | 1.08 (1.07-1.10) | <0.001 |  |
| PEEP (cmH_2_O) | 1.37 (1.33-1.41) | <0.001 | MBP (mmHg) | | | 0.99 (0.99-1.00) | <0.001 |  |
| Pplat (cmH_2_O) | 1.14 (1.12-1.16) | <0.001 | SPO_2_ (%) | | | 0.91 (0.89-0.94) | <0.001 |  |
| BMI - body mass index, SOFA - sequential organ failure assessment, SAPS II - simplified acute physiology score II, COPD - chronic obstructive pulmonary disease, SBT - spontaneous breathing trial, VT - tidal volume, RR - respiratory rate, PEEP - positive end expiratory pressure, Pplat - plateau pressure, Ppeak - peak inspiratory pressure, ΔPaw - dynamic driving pressure, Cdyn - dynamic lung compliance, MP - mechanical power, Cdyn-MP - MP normalized to dynamic lung compliance, FiO_2_ - inspired oxygen concentration, WBC - white blood cell count, PLT - platelets, Hb - hemoglobin, SCr - serum creatinine, Uorate - urine output per hour, HR - heart rate, BF - breathing frequency during SBT, MBP - mean blood pressure, SPO_2_ - pulse oximetry. | | | | | | | |  |

| **S3 Table. Association between Cdyn-MP and weaning failure in complete cases.** | | | | | | | | | | | | |
| --- | --- | --- | --- | --- | --- | --- | --- | --- | --- | --- | --- | --- |
| **Variables** | **Unadjusted** | | |  | **Model 1** | |  | **Model 2** | |  | **Model 3** | |
|  | **OR (95% CI)** | ***P* value** |  | **OR (95% CI)** | | ***P* value** |  | **OR (95% CI)** | ***P* value** |  | **OR (95% CI)** | ***P* value** |
| Cdyn-MP (per100J/min  ×cmH_2_O/ml×10^-3^) | 1.33 (1.29-1.38) | < 0.001 |  | 1.32 (1.28-1.37) | | < 0.001 |  | 1.23 (1.17-1.28) | < 0.001 |  | 1.23 (1.18-1.28) | < 0.001 |
| Q1 (n=519, ≤164.9) | 1 (Ref) |  |  | 1 (Ref) | |  |  | 1(Ref) |  |  | 1(Ref) |  |
| Q2 (n=520, 164.9-337.4) | 5.96 (4.19-8.5) | < 0.001 |  | 5.91 (4.14-8.43) | | < 0.001 |  | 4.90 (3.41-7.04) | < 0.001 |  | 4.90 (3.41-7.05) | < 0.001 |
| Q3 (n=519, 337.4-566.4) | 11.69 (8.23-16.6) | < 0.001 |  | 11.49 (8.08-16.34) | | < 0.001 |  | 7.96 (5.52-11.47) | < 0.001 |  | 7.91 (5.48-11.41) | < 0.001 |
| Q4 (n=520, ≥566.4) | 19.73 (13.83-28.13) | < 0.001 |  | 18.4 (12.85-26.36) | | < 0.001 |  | 8.57 (5.68-12.93) | < 0.001 |  | 8.63 (5.71-13.05) | < 0.001 |
| P for trend |  | < 0.001 |  |  | | < 0.001 |  |  | < 0.001 |  |  | < 0.001 |
| OR - odds ratio, CI - confidence interval, Cdyn-MP - mechanical power normalized to dynamic lung compliance, Q1, Q2, Q3, and Q4 are quartiles of the Cdyn-MP. Model 1 was adjusted for age, BMI and SOFA score. Model 2 was adjusted for Model 1+RR, PEEP, Pplat and FiO_2_. Model 3 was adjusted for Model 2+WBC, SCr, Uorate, HR, MBP and SpO_2_. | | | | | | | | | | | | |

**S4 Table. Predictability of related parameters for weaning failure occurrence in mechanically ventilated patients.**

| **Variables** | **AUC**  **(95% CI)** | ***P* value** | **Thresholds** | **Sensitivity** | **Specificity** | **PPV** | **NPV** |
| --- | --- | --- | --- | --- | --- | --- | --- |
| PEEP (cmH_2_O) | 0.712 (0.695-0.729) | <0.001 | >5.4 | 0.59 | 0.75 | 0.74 | 0.60 |
| ΔPaw (cmH_2_O) | 0.667 (0.650-0.684) | <0.001 | >12.7 | 0.70 | 0.57 | 0.75 | 0.50 |
| Cdyn (ml/cmH_2_O) | 0.689 (0.672-0.706) | <0.001 | <31.3 | 0.57 | 0.67 | 0.71 | 0.52 |
| MP (J/min) | 0.745 (0.730-0.761) | <0.001 | >11.3 | 0.71 | 0.65 | 0.56 | 0.78 |
| Cdyn-MP (J/min×cmH_2_O/ml×10^−3^) | 0.760 (0.745-0.776) | <0.001 | >317.8 | 0.76 | 0.63 | 0.56 | 0.81 |
| AUC - area under the curve, PPV - Positive Predictive Value, NPV - negative predictive value, PEEP - positive end expiratory pressure, ΔPaw - dynamic driving pressure, Cdyn - dynamic lung compliance, MP - mechanical power, Cdyn-MP - MP normalized to dynamic lung ompliance. | | | | | | | |

**S1** **Fig. Flowchart showing the screening process used in the study.**

**S2 Fig. Relationship between Cdyn-MP and weaning failure in mechanically ventilated patients based on basic features when analyzing Cdyn-MP as quartiles.** Each stratification was adjusted for age, body mass index, SOFA score, respiratory rate, positive end-expiratory pressure, plateau pressure, FiO_2_, white blood cell count, serum creatinine, Uorate, heart rate, mean blood pressure and SpO_2_ except the stratification factor itself. Squares indicate odds ratios (ORs), with horizontal lines indicating 95% confidence intervals.


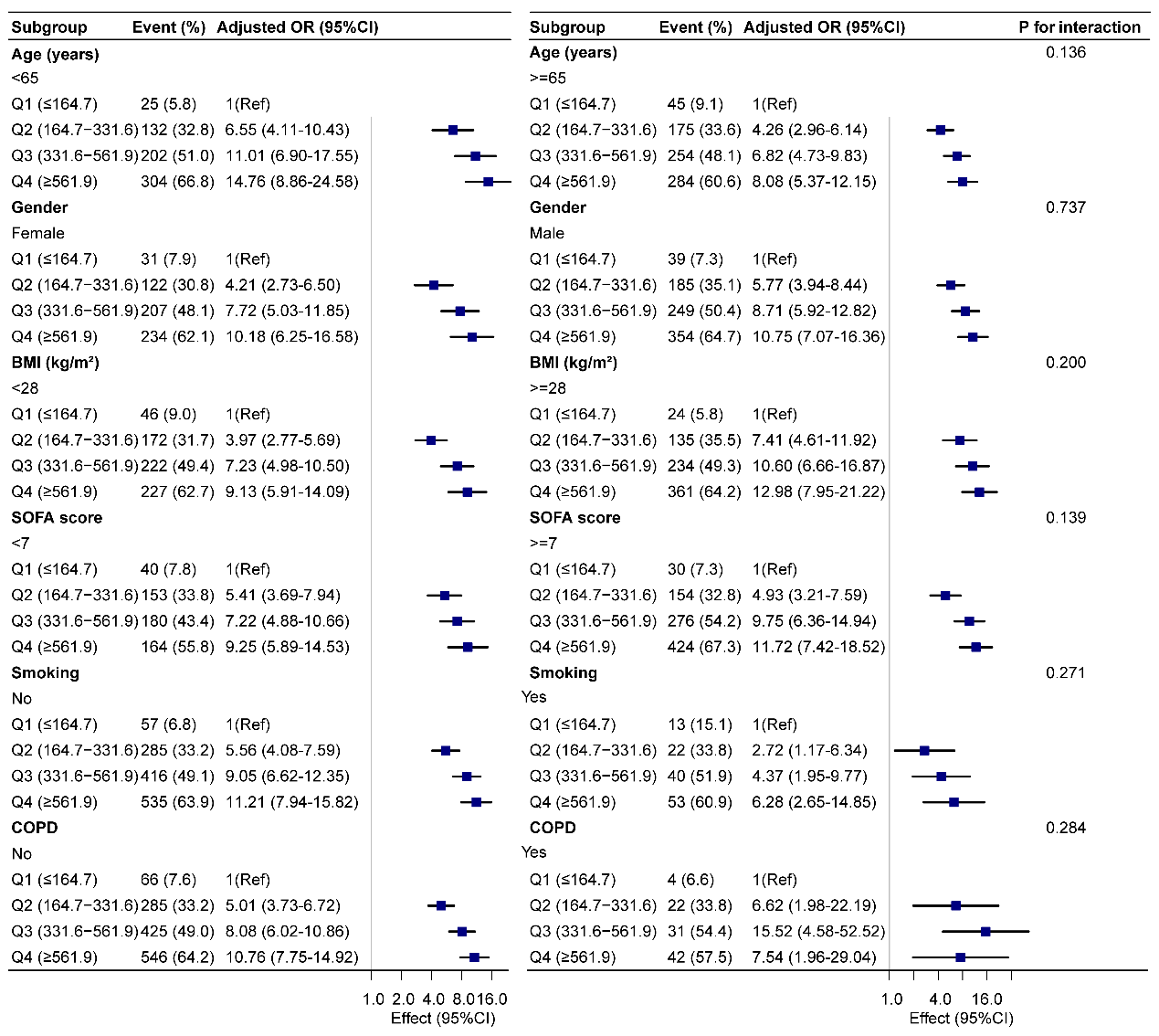


**S3 Fig. Association between mechanical power normalized to dynamic lung compliance (Cdyn-MP) and weaning failure in mechanically ventilated patients with different comorbidities. (A) Congestive heart failure. (B) Chronic kidney disease. (C) Diabetes. (D) Stroke.** Solid lines represent the best-fit curve, and dashed lines represent a 95% confidence interval. Odd ratios (ORs) were adjusted for age, body mass index, SOFA score, respiratory rate, positive end-expiratory pressure, plateau pressure, FiO_2_, white blood cell count, serum creatinine, Uorate, heart rate, mean blood pressure and SpO_2_.


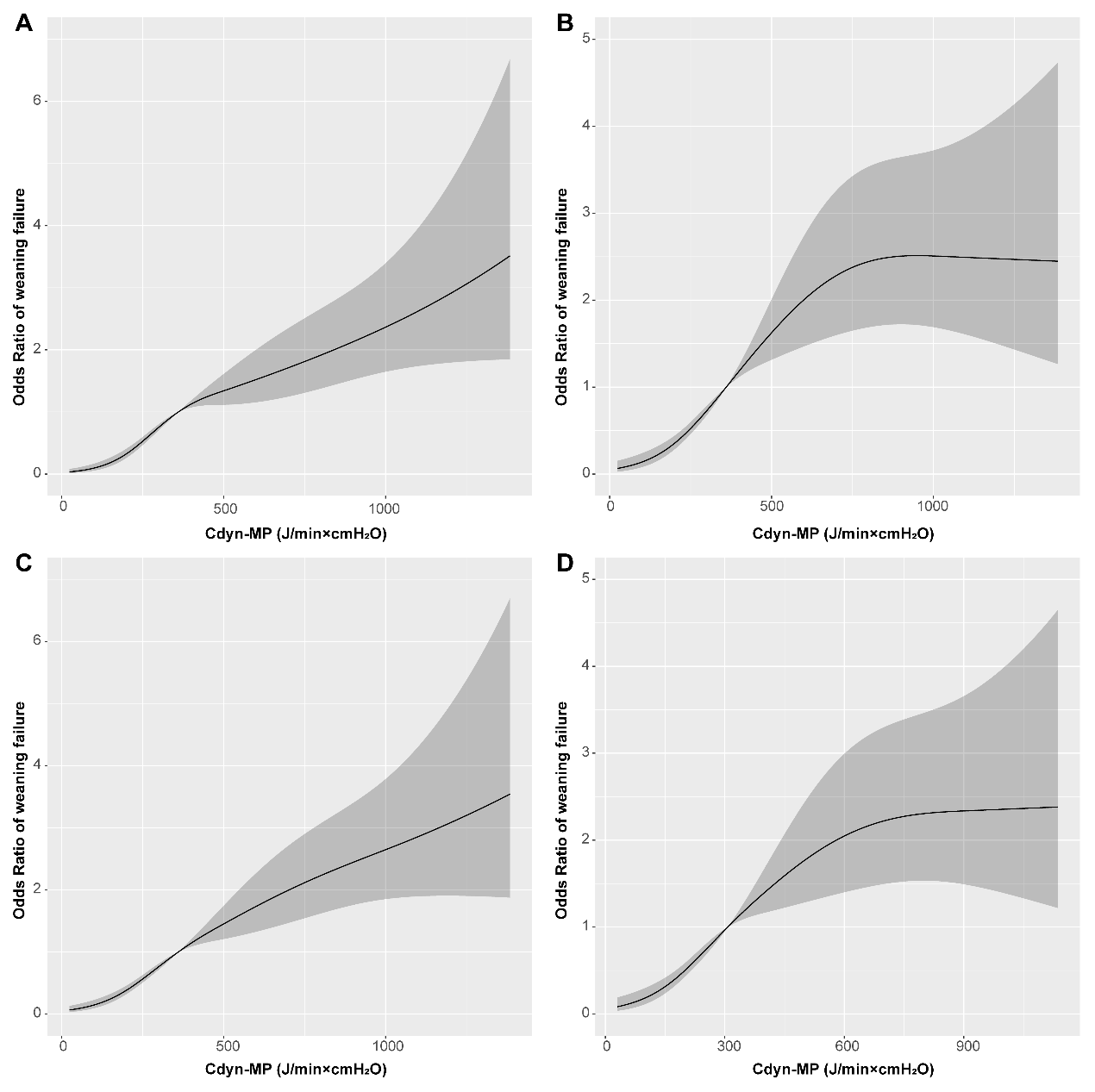

Supplement: S1 File — (DOCX) [file pone.0306116.s002.docx]
